# The Treatment of Whooping-Cough

**Published:** 1910-01-22

**Authors:** C. Willett Cunnington


### The General Practitioner's Column.

[Contributions to this Column are invited, and, if accepted, will be paid for.]

### THE TREATMENT OF WHOOPING-COUGH.

By C. WILLETT CUNNINGTON, M.B., B.C.Cantab.

For the purpose of treatment cases of this disease may conveniently be divided into two groups—those cases where the child is best treated by being allowed to be up and about, that is, the more chronic cases; and those where the child is necessarily confined to bed, and these are the more acute cases. Pertussis is a disease for which a large number of drugs are recommended. Each man has a favourite upon which he is apt to rely, whether from experience or fancy. Many such specifics are useless, perhaps even injurious; nevertheless it is helpful to compare notes with other practitioners and to hear what means they prefer to employ in combating a disease so frequent and occasionally so dangerous as whooping-cough.

It must be remembered that most of the fatal cases are culled from hospital clinics, the patients being drawn from the most unhealthy surroundings. Nevertheless in better classes alarming cases are common enough to merit careful attention to the treatment.

In the subacute and chronic cases we have to consider chiefly matters of hygiene. In other words, we endeavour to prevent relapses and to shorten the course of a disease which is only too frequently most tedious; anything we are able to do in this direction will earn the gratitude of the child's parents.

There can be no question that abundance of fresh air, particularly sea air, helps to shorten the course. One should advise a bracing sea side, and that the patient be turned out of doors all day and every day, utilising all the sunshine available. Parents sometimes hesitate to do this, fearing lest bronchitis may be set up. If the lungs are free from moist râles, especially at the bases, the risk is very slight, and worth running, provided a careless nursemaid is not allowed to leave a child standing about in a keen east wind. It is never wise to send a child out into unsheltered places if there are râles in the chest.

In chronic cases there is one further danger which has to be borne in mind when vomiting is frequent. The child's vitality may suffer seriously from simple loss of food due to the sickness, and this although he is up and playing in the nursery or even out of doors. It is curious how a mother will fail to realise that her child is being half-starved by vomiting soon after meals. One suspects that tuberculosis, recognised as a fairly common sequela to whooping-cough, may be induced by the inanition kept up in this way. The mother should be warned on this point; a simple rule is that the child should always be given food a few minutes after each attack of vomiting.

It is well, too, in these long drawn-out cases of whooping-cough to have the child weighed weekly. Medicinally there is perhaps nothing better than some preparation of cod-liver oil combined with malt. An old-fashioned drug, found by experience to diminish the number of paroxysms, is benzol in minim doses, made up as an emulsion with syrup. To prevent re-infection of the patient and infection of other children all articles contaminated by vomit or nasal secretions should be disinfected as speedily as possible.

To turn now to the acute type of case. The clinical picture is that of a child suffering from acute bronchitis or bronchiolitis, the symptoms being combined with the familiar paroxysms, the whoop followed by vomiting. It is well to bear in mind that there are two factors at work against the patient's strength, one being the mechanical strain produced by the paroxysm, and the other the toxæmia due to the poison of the specific bacillus of the disease.

Considering the former alone for a moment, it is needful to remember that the already existing respiratory embarrassment is throwing a strain on the right chambers of the heart; add to this the sudden stress produced by the paroxysmal whoop and we see at once that a further load is thrust upon the same organ, already labouring under difficulties. The effects are made manifest in the resulting cyanosis, and œdema of eyelids and face. The mechanical factor, then, leads to a state of affairs which may be regarded as an acute attack of dilatation of the right auricle and ventricle, and the condition must be treated as such. It is suggestive that in fatal cases of pertussis the post-mortem shows invariably dilatation of the right heart.

If this fact be kept constantly in mind the lines of appropriate treatment will be clear. Strychnine, digitalis, and oxygen are the types of drugs called for. Occasionally in bad cases where the dyspnœa is extreme, strychnine has to be given hypodermically and pushed without fear. Injections of camphor are useful. The effect of oxygen is quite remarkable where there is much cyanosis, but it is desirable that the gas should be passed through warm water in a wash-bottle before reaching the patient's mouth, as a stream of cold dry gas is likely to set up paroxysms of whooping. A good word may be inserted here for leeches, even with quite young patients, to relieve extreme distension of the right heart. In speaking of the use of drugs one must remember that many, theoretically useful, become difficult to exhibit by the mouth on account of their disagreeable taste, and a child with bad whooping-cough will not tolerate coercion without suffering an increase of the paroxysms at each attempt. The hypodermic syringe will generally meet this difficulty.

Let us now consider the effects produced by the toxæmia, setting aside for the moment the mechanical results of the paroxysms. As a matter of clinical experience we not infrequently have cases where the toxæmia is severe and the number of paroxysms few; indeed, the child may scarcely whoop at all, yet be exceedingly ill. The temperature is high, the respirations rapid, and the patient gives the picture of broncho-pneumonia. But on examination there may be but few physical signs in the chest. The cardiac dulness is much increased on the right side. The poison of the disease seems to pick out especially the myocardium, and when the effects of the toxic and mechanical factors are acting together it is clear that the patient's life depends practically on the powers of resistance of the right side of the heart. Fortunately a child's myocardium is astonishingly resilient, and there seems to be no limit of stretching from which it cannot recover.

In treating a severe case it is not enough to suppress the paroxysms; we must remember that the toxæmia may by itself kill the patient. To check the former antipyrin is advised, in doses of a grain per year of age, every three hours; but this drug, and the same applies to bromides, is a cardiac depressant. Belladonna is a cardiac stimulant, and if given in doses sufficient to produce physiological signs diminishes the frequency of the whoop, and also supports the heart. Where there is much excitement and sleeplessness opium is very valuable, provided there is not too much bronchitis.

A cresoline lamp is a useful addition, but it must be used with intelligence. The vapour acts partly as a disinfectant, partly as a sedative to the mucous membrane of the trachea and bronchi. To get its full effect the lamp should be burnt for an hour or two with the windows of the room closed. Then the lamp may be extinguished and the windows opened for a few minutes to get the atmosphere thoroughly changed. To keep windows open while the lamp is alight is to counteract its antiseptic effect.

Feeding in severe cases is generally a difficulty. There can be nothing better than milk peptonised to promote speedy assimilation. The feeds should be small in amount, and frequent, and they should be repeated after each attack of vomiting, if this is a troublesome event.

The difficulties of a case are naturally much increased by the presence of pneumonia, bronchitis, and the like. Accidental complications, such as hæmorrhage into various organs, can hardly be guarded against beyond endeavouring to check the paroxysms in the ways mentioned.

Care must be exercised in allowing a child to get up after the acute stage is passed. Relapses are common and can be induced by emotional disturbances. Further, it may be noted that the child has been of necessity much "spoiled" during the acute stage, and to reinstate discipline in the nursery is a delicate matter. The child remains for some time in a highly emotional state, and any rebuke is apt to induce whooping.

